# Patterns of pediatric injuries: a prospective cross-sectional study at the Tamale Teaching Hospital, Tamale, Ghana

**DOI:** 10.11604/pamj.2025.50.52.42929

**Published:** 2025-02-13

**Authors:** Emmanuel Yeboah Gyabaah, Hezron Bondzie, John Abanga Alatiiga, Charles Mock

**Affiliations:** 1 School of Medicine, University for Development Studies, Tamale, Ghana,; 2 Department of Surgery, Tamale Teaching Hospital, Tamale, Ghana,; 3 Harborview Medical Center, University of Washington, Seattle, Washington, United States of America

**Keywords:** Pediatric injuries, epidemiology, developing countries, trauma, Ghana

## Abstract

**Introduction:**

childhood injuries account for more deaths than all other diseases combined in children between the ages of 5-14. Despite underreporting, morbidity and mortality from injuries are highest in low- and middle-income countries. Data on injury patterns can influence policy and help implement preventive measures. This study aims to determine the pattern of pediatric injuries at the Tamale Teaching Hospital.

**Methods:**

data were prospectively collected from February 2023 to August 2023 at the Tamale Teaching Hospital on all patients below 17 years who sustained injuries resulting in admission for at least 24 hours. Injury severity was graded using the Kampala and Pediatric Trauma Scores. Data on patient demography, type of injury, mechanism of injury, treatment, and mortality were collected and analyzed.

**Results:**

of 147 participants, 96 were males, with a mean age of 7.9 (± 4.8) years. The top three injuries were isolated head injuries (n=62, 42.2% of all patients), isolated fractures (n=25, 17%) and isolated burns (n=18, 12.2%). There were 12 (8.2%) participants with multiple injuries. Isolated head injuries were the most common type of injury for all ages above one year. The majority (n=110, 62.6%) was treated conservatively.

**Conclusion:**

commonest injury patterns among children at the Tamale Teaching Hospital are head injuries, fractures, and burns. Improvements in capacity for neurosurgery and burn care should be prioritized.

## Introduction

Worldwide, trauma has been neglected on the public health scene despite overwhelming evidence of its impact [1-3]. According to the Lancet Commission on Global surgery, road traffic accidents (RTAs) together with other non-communicable diseases are expected to surpass the burden of communicable diseases within the next 20 years [4]. Amputations due to traumatic causes are on a steady rise year after year [5] and RTA is already the leading cause of pediatric admissions in low-and-middle-income countries (LMICs) [6,7]. Globally, childhood injuries account for more deaths than all other diseases combined in children between the ages of 5-14 [8]. In the under-18 age population, potential life lost attributable to death from injuries surpasses death resulting from cancer and infectious diseases combined [9]. One (1) out of every 4 children is involved in an unintentional injury that requires medical attention each year [10]. Trauma is the most common cause of death in children older than 5 and remains so until about 40 years of life [11]. Morbidity and mortality due to trauma are highest in LMICs [12-14]. This may even be underreported because of poor emergency and prehospital care services [15]. Children who survive traumatic events may require expert care for life [9].

Long hospital stays and lifelong morbidity can have a significant strain on a child´s social development and education. Even in high-income countries (HICs), medical expenses can become unbearable. There is a need for government cooperation in terms of policies and funding [2,12], yet governments in LMICs have been largely uncooperative and unwilling [2,16]. Due to the paucity of data, the burden of injury in children can be difficult to accurately determine, and evidenced-based policymaking is hindered. By implementing appropriate preventive measures specific to locations, a majority of trauma incidents can be prevented. The major means of transportation in Tamale are motorcycles and tricycles, on poor road networks. This makes Tamale a high-risk area for injuries. To the best of our knowledge, there is currently no published data on pediatric injuries from the Tamale Teaching Hospital. This study aimed to assess the patterns of pediatric injuries at the Tamale Teaching Hospital and to identify priority areas to strengthen care for injured children at the hospital.

## Methods

**Study design and setting:** the study was a 6-month prospective cross-sectional study carried out at the emergency department of Tamale Teaching Hospital in the Northern Region of Ghana. Tamale Teaching Hospital is a tertiary facility and a major trauma referral center for Tamale metropolis and beyond. Thus, cases from all 5 northern regions refer to the facility. Data were collected for a period of 6 months. From February 14, 2023 to August 14, 2023.

**Study population:** target population were all injured children admitted to the Tamale Teaching Hospital. Inclusion criteria were children below the age of 17 who sustained injuries resulting in admission for at least 24 hours, and/or death. Exclusion criteria were admission for less than 24 hours. All pediatric trauma patients admitted during the 6-month period (February 14 to August 14, 2023) were sampled.

**Data collection:** a two-page structured questionnaire with open and closed-ended questions was developed for this study and was used to guide interviews. Interviews took approximately 15 minutes. Data on age, sex, the mechanism of injury, the type of injury, treatment, and mortality were collected. Additional data were accessed from patients' medical records where necessary.

**Data analysis:** data were analyzed using IBM SPSS statistics software (version 25). Demographics were categorized as sex (male, female) and age (<1 year, 1-5 years, 6-10 years, and 11-16 years). Participants were classified into various injury severity groups using the Kampala Trauma Score (KTS) and the Paediatric Trauma Score (PTS). Both scores assign numeric scores reflecting the severity of all injuries. In our analysis, scores were grouped into categories of injury severity: mild, moderate, and severe. Mechanism of injury was divided into RTA, assault, falls, burns, sports injuries, and others. RTA included accidents involving car-to-car, motorcycle-to-motorcycle, car-to-motorcycle, pedestrians, and other powered two-and-three wheelers. Falls include falls from heights and from running or standing. Sports injuries included those sustained from play. Others included all others that did not fit previous descriptions. Type of injury was broadly classified as fractures, head injuries, abdominal injuries, soft tissue injuries including burns, chest injuries, and others. Fractures were further classified as either open or closed and specific bones involved. Head injuries were also subdivided into skull fractures, diffuse lesions, focal lesions, and lacerations. Data analysis included the derivation of descriptive statistics, including frequencies and percentages.

**Ethical consideration:** the authors gained written informed consent from parents or guardians for all participants. Ethical clearance for the study was gained from the Committee on Human Research Publication and Ethics of the Kwame Nkrumah University of Science Technology with reference number CHRPE/AP/793/22.

## Results

**General characteristics of the study population:** a total of 147 participants were included in the study. Most were males. Participants were equally divided across the age groups (Table 1). The mean age of all participants was 7.9 (± 4.8). The Kampala Trauma Score (KTS) classified most participants as moderate, whereas the PTS classified most participants as minor. Majority of the injuries resulted from RTAs, followed by falls and then burns (Table 1).

**Table 1:** participant characteristics

Age groups (Years)	Frequency (147)	Percentage (%)
<1	2	1.4
1 – 5	48	32.6
6 – 10	49	33.3
11 – 16	48	32.7
Total	147	100
**Sex**		
Male	96	65.3
Female	51	34.7
**Mechanism of injury**		
RTA*	70	47.6
Falls	41	27.9
Burns	18	12.2
Sport injuries	3	2.0
Assault	2	1.4
Others	13	8.8
Severity score (KTS**/PTS***)		
Mild	38/71	25.9/48.3
Moderate	70/60	47.6/40.8
Severe	39/16	26.5/10.9

* RTA: road traffic accident;**KTS: Kampala trauma score;***PTS: pediatric trauma score

**Figure 1:**
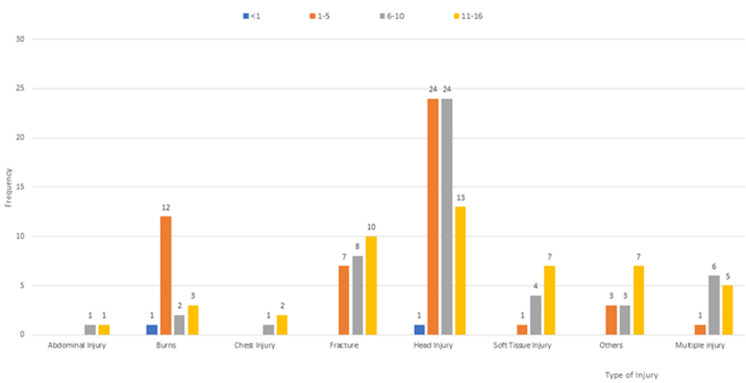
distribution of types of injuries within age groups

**Types of injuries:** the commonest type of injury was a head injury (n=71), with skull fractures being the commonest subtype. Out of the 71 participants with head injuries, 62 had an isolated head injury, eight had a head injury and a fracture, and one had a head injury and an abdominal injury. The second most common injury was fracture (n=37). Twelve (12) participants had a fracture and at least one other injury (Table 2). Fourteen (14) sustained fractures in multiple bones (Table 2). Upper limb fractures were common than lower limb fractures. Burns were the third most common injury (n=18) (Table 2). Isolated head injuries were the most common injuries for most age groups (Figure 1). The second most common injury in each age group was: burns for 1-5 year old; and isolated fractures for those between 6-16 years. There were only two children under one year, one suffered from head injuries and one from burn injuries (Figure 1).

**Table 2:** types of injury and treatments received by participants

Type of Injury	Frequency (147)	Percentage (100%)	Treatment			
			Surgery	Percentage (100%)	Conservative	Percentage (100%)
Fractures*						
Open	9	36.0	6	66.8	3	33.3
Closed	16	64.0	5	31.2	11	68.8
**TOTAL**	25	17.0	11	44.0	14	56.0
Head Injuries*						
Lacerations	11	17.7	0	0.0	11	100.0
Skull fractures	25	40.3	11	44.0	14	56.0
Diffuse lesions	14	22.6	0	0.0	14	100.0
Focal lesions	12	19.4	3	25.0	9	75.0
**TOTAL**	62	42.2	14	22.6	48	77.4
Burns	18	12.2	3	16.7	15	83.3
Soft tissue injury	12	8.2	4	33.3	8	66.7
Chest injuries	3	2.0	0	0.0	3	100.0
Abdominal injury	2	1.4	1	50.0	1	50.0
Others	13	8.8	3	23.1	10	76.9
Multiple injuries**	12	8.2	1	8.3	11	91.7
**TOTAL OF ALL**	147	100%	37	25.2	110	74.8
Bone Involved***						
**Upper limb**						
Radius	10	18.9	3	30.0	7	70.0
Ulnar	8	15.1	3	37.5	5	62.5
Humerus	5	9.4	2	40.0	3	60.0
Hands	4	7.6	2	50.0	2	50.0
Clavicle	1	1.9	0	0.0	1	100.0
**Lower limb**						
Tibia	9	17.0	2	25.0	7	75.0
Fibular	6	11.3	1	16.7	5	83.3
Feet	5	9.4	2	40.0	3	60.0
Femur	5	9.4	3	60.0	2	40.0

* Percentages within the groups fractures and head injuries indicate percentages within those groups; **All 12 had fractures and at least another injury; 8 had fractures and head injuries. One (1) had a fracture, a head injury and an abdominal injury (hemoperitoneum); two (2) had a fracture and a dislocation, and one (1) had a fracture and a chest injury (hemothorax); ***14 participants had fractures of multiple bones. Hence, the total number of bones involved adds to more than the 37 participants with fractures. Percentages for specific bones based on a total number of fractures (n=53); fractures of hands and feet counted as one fracture, even if multiple bones were involved (e.g. multiple carpals and metacarpals).

**Treatment and outcome of injuries:** majority were treated conservatively (Table 2). Three out of 9 patients with open fractures presented early with puncture wounds and minimal contamination and were treated conservatively. Fifteen out of the 18 patients with burns were treated conservatively due to limited burn management capacity, including limited availability of dermatomes and meshing equipment. Some of these were later referred to other facilities for surgical management. There were 42 operations performed (some patients having more than one operation). Types of operations performed included: orthopedic procedures (n=17), elevation of depressed skull fractures (9), evacuation of intracranial hematoma (5), skin grafting or other plastic surgery procedures (5), amputations (4), laparotomy (1), and other (1). A total of three mortalities were recorded. These were from burns (n=2) and head injury (n=1). Table 3 shows the relationship between severity of injury and mortality.

**Table 3:** Kampala trauma scores and paediatric trauma scores against mortality

	Number of patients	Percentage (%)	Mortality	Percentage (%)
**Kampala trauma score**				
9-10 (Mild)	38	25.9	1	2.6
7-8 (Moderate)	70	47.6	1	1.4
<6 (Severe)	39	26.5	1	2.5
**TOTAL**	147	100	3	2.0
**Pediatric trauma Score**				
9-12 (Minor trauma)	71	48.3	1	1.4
6-8 (Potentially life-threatening)	60	40.8	2	3.2
0-5 (Life-threatening)	16	10.9	0	0.0
**TOTAL**	147	100	3	2.0

## Discussion

This study aimed to outline the patterns of injuries occurring among children at the Tamale Teaching Hospital and to assess priority areas for strengthening trauma care among children in the hospital. Head injuries were the most common injury observed, with the majority resulting from RTA. Fractures were the second most common followed by burns. Majority were treated conservatively and three (3) mortalities were recorded. The total number of 147 participants in the 6-month period was fewer than what has been recorded in other parts of the country [16]. This may be due to poor health-seeking behaviors of people in this part of the country and not necessarily low rates of injury. Participants were evenly distributed across all age groups except in the under-one age group which recorded the lowest frequency (1.4%). This may be attributed to the low level of activity within the under-one age group, and hence being less likely to sustain injuries compared to children in other age groups. The male preponderance observed (in low-and-middle income countries: 1) is consistent with literature from other geographical locations [16-18]. A systematic review of pediatric head injuries in Africa showed the most common type of injury to be skull fractures with 28.3% of participants in that study sustaining a skull fracture [19]. In the current study, skull fractures were the most common head injury, but with a higher frequency (40.3% of all head injuries). Although head injuries were the most common reason for trauma admissions in children, there was only one neurosurgeon for both pediatric and adult populations in Tamale Teaching Hospital (TTH) and the whole of northern Ghana at the time of this study.

An increase in neurosurgical capacity should therefore be prioritized. Fractures were the second most common injury in the current study. The most common type of fracture was a forearm (radius or ulna) fracture (34.0%), followed by leg (tibia or fibula) fracture (27.7%). Guifo *et al*. report a similar pattern in Cameroon [20] but report a higher number of femoral fractures (16%) compared with the 8.5% from our study. Care of fractures in the current study seemed appropriate in terms of the balance of operative and conservative treatment. There are five orthopedic trauma surgeons at TTH. The overall capacity for orthopedic care appears adequate. Soft tissue injuries included bruises, wounds, skin lacerations, and burns. Burns accounted for 66.7% of all soft tissue injuries, and 13.6% of all pediatric injuries with the majority (65.0%) being ≤5 years. This figure is consistent with what has been reported in the UK [21] and sub-Saharan Africa [22]. In the current study, only a small percentage of burn patients received operative care (e.g. skin grafts), indicating that improvements in the capacity for skin grafts should be prioritized.

Cintean *et al*. reported that pediatric injuries are usually minor and do not necessitate surgery [17]. Our study confirms this observation with the majority (62.6%) being treated conservatively. One of the three patients who died suffered from a head injury and the other two died from burns. The patient who died from a head injury was classified as moderate by the KTS. Similar findings of the KTS labeling patients as mild or moderate who later on died have been reported in the literature [23] and the utility of the KTS as a tool for individualized management of patients in the emergency department has been questioned [24]. These studies were however in patients older than 18 years and there is insufficient data for comparison in the pediatric population. The PTS also classified the patient who died from a head injury as mild. The numbers are however too small to make conclusions about the accuracy of KTS or PTS for prediction of mortality. Further studies are needed to determine the effectiveness of these scores in predicting mortality in children with head injuries. This study has provided data from a tertiary referral facility on pediatric injuries in a previously unstudied population and has identified areas for improvement in pediatric trauma care. Although most of the findings are in agreement with data from other locations, the generalizability of the study is limited as this is a single-center study. Again, only children who reported to the emergency department were included in the study. Children who were treated in the clinic or by traditional healers and bonesetters, rampant in this area, were not captured in the study.

## Conclusion

The leading types of injuries were head injuries, fractures, and burns. More than a third of all fractures involved multiple bones, while less than a quarter of all injuries involved multiple organ systems. Injuries were more common among males and evenly distributed among age groups. The majority of injuries resulted from RTAs, falls, and burns. A greater proportion of the injuries are managed conservatively. Improving capabilities for burns and neurosurgical care should be the focus of efforts to strengthen trauma care at the Tamale Teaching Hospital.

### What is known about this topic

- Morbidity and mortality from childhood injuries are highest in low-and-middle income countries despite underreporting;
- Knowledge of patterns of injuries can help implement preventive measures;
- There is currently no data on paediatric injuries from the Tamale Teaching Hospital in northern Ghana.

### What this study adds

- Head injuries are the most common type of injury among children at the Tamale Teaching Hospital, followed by fractures and burns;
- Majority of injuries among children are treated conservatively with good outcomes;
- This study has identified neurosurgical and burns care as priority areas for improvement for paediatric trauma care at the hospital.

## References

[1] Mhando S, Lyamuya S, Lakhoo K (2006). Challenges in developing paediatric surgery in Sub-Saharan Africa. Pediatr Surg Int.

[2] Ademuyiwa AO, Usang UE, Oluwadiya KS, Ogunlana DI, Glover-Addy H, Bode CO (2012). Pediatric trauma in sub-Saharan Africa: Challenges in overcoming the scourge. J Emerg Trauma Shock.

[3] Reading R, Bissell S, Goldhagen J, Harwin J, Masson J, Moynihan S (2009). Promotion of children´s rights and prevention of child maltreatment. Lancet.

[4] Meara JG, Leather AJM, Hagander L, Alkire BC, Alonso N, Ameh EA (2015). Global Surgery 2030: Evidence and solutions for achieving health, welfare, and economic development. Lancet.

[5] Yempabe T, Salisu WJ, Buunaaim ADB, Hussein H, Mock CN (2021). Epidemiology of surgical amputations in Tamale teaching hospital, Ghana. Journal of Medical and Biomedical Sciences.

[6] Sharma M, Lahoti B, Khandelwal G, Mathur R, Sharma S, Laddha A (2011). Epidemiological trends of pediatric trauma: A single-center study of 791 patients. J Indian Assoc Pediatr Surg.

[7] Bradshaw CJ, Lakhoo K, Ameh E, Banu T, Borgstein E, Croaker D (2016). A day in the life of a paediatric surgeon: a PAPSA research study. Ann Pediatr Surg.

[8] Krug EG, Sharma GK, Lozano R (2000). The global burden of injuries. Am J Public Health.

[9] Hennrikus WL, Sarwark JF, Esposito PW, Gabriel KR, Guidera KJ, Roye DP (2008). Management of pediatric trauma. Pediatrics.

[10] Danseco ER, Miller TR, Spicer RS (2000). Incidence and costs of 1987-1994 childhood injuries: demographic breakdowns. Pediatrics.

[11] Bickler SW, Rode H (2002). Surgical services for children in developing countries. Bull World Health Organ.

[12] Mock C, Cherian MN (2008). The global burden of musculoskeletal injuries: challenges and solutions. Clin Orthop Relat Res.

[13] Meel BL (2003). Mortality of children in the Transkei Region of South Africa. Am J Forensic Med Pathol.

[14] Bradshaw CJ, Bandi AS, Muktar Z, Hasan MA, Chowdhury TK, Banu T (2018). International Study of the Epidemiology of Paediatric Trauma: PAPSA Research Study. World J Surg.

[15] Suryanto Plummer V, Boyle M (2017). EMS systems in lower-middle income countries: a literature review. Prehosp Disaster Med.

[16] Abantanga FA, Mock CN (1998). Childhood injuries in an urban area of Ghana a hospital-based study of 677 cases. Pediatr Surg Int.

[17] Cintean R, Eickhoff A, Zieger J, Gebhard F, Schütze K (2023). Epidemiology, patterns, and mechanisms of pediatric trauma: a review of 12,508 patients. Eur J Trauma Emerg Surg.

[18] Aoki M, Abe T, Saitoh D, Oshima K (2019). Epidemiology, patterns of treatment, and mortality of pediatric trauma patients in Japan. Sci Rep.

[19] Gupta N, Kasula V, Waguia Kouam R, Seas A, Esene I, Malomo AO (2023). Management and outcomes of pediatric traumatic brain injury in Africa: a systematic review. J Neurosurg Pediatr.

[20] Guifo ML, Tochie JN, Oumarou BN, Tapouh JRM, Bang AG, Ndoumbe A (2017). Paediatric fractures in a sub-saharan tertiary care center: a cohort analysis of demographic characteristics, clinical presentation, therapeutic patterns and outcomes. Pan Afr Med J.

[21] Kemp AM, Jones S, Lawson Z, Maguire SA (2014). Patterns of burns and scalds in children. Arch Dis Child.

[22] Nthumba PM (2016). Burns in sub-Saharan Africa: a review. Burns.

[23] Ariaka H, Kiryabwire J, Hussein S, Ogwal A, Nkonge E, Oyania F (2020). A Comparison of the Predictive Value of the Glasgow Coma Scale and the Kampala Trauma Score for Mortality and Length of Hospital Stay in Head Injury Patients at a Tertiary Hospital in Uganda: A Diagnostic Prospective Study. Surg Res Pract.

[24] Macleod J, Kobusingye O, Frost C, Lett R (2007). Kampala Trauma Score (KTS): is it a new triage tool?. ECAJS.

